# Feature Analysis for Evaluating the Risk of Postoperative Delirium in Pediatric Patients

**DOI:** 10.3390/jcm15051892

**Published:** 2026-03-02

**Authors:** Andrzej Czyrski, Jowita Rosada-Kurasińska, Weronika Ziętkiewicz, Klaudia Sarniak, Estera Szwedziak, Agnieszka Bienert, Alicja Bartkowska-Śniatkowska

**Affiliations:** 1 Poznan University of Medical Sciences, Department of Physical Pharmacy and Pharmacokinetics, Rokietnicka 3 Street, 60-806 Poznań, Poland; 2 Poznan University of Medical Sciences, Department of Paediatric Anaesthesiology and Intensive Therapy, Szpitalna 27/33 Street, 60-572 Poznań, Poland; 3 Poznan University of Medical Sciences, Adult Psychiatry Clinic, Department of Psychiatry, Szpitalna 27/33 Street, 60-572 Poznań, Poland

**Keywords:** CAPD scale, PAED scale, postoperative delirium, pediatric patients, postoperative pain, behavioral changes

## Abstract

**Background/Objectives:** Postoperative delirium is an acute condition commonly seen in pediatric patients. It is often observed in intensive care units for patients undergoing general anesthesia. Characteristic symptoms include disturbances of consciousness, attention, perception, or disorientation. The occurrence of delirium can be assessed using the CAPD and PAED scales. **Methods:** A single-center observational cohort studyof the 2022–2024 results was conducted. A total of 89 patients of the Pediatric Anesthesiology and Intensive Care Unit undergoing procedures under general anesthesia were included in this study. The state of delirium just after the recovery from anesthesia was assessed using the CAPD and PAED scales. **Results:** A total of 60% of patients experienced delirium taking the results according to the CAPD scale, 39% according to the PAED scale. A score indicating delirium according to both the CAPD and PAED scales was recorded in 21% of the patients examined. The results of the correlation analyses indicate a strong relationship between the CAPD and PAED scales. The feature analysis indicated there was a correlation between the occurrence of delirium and chronic disease. One of the confirmed risk factors for the development of postoperative delirium in children is postoperative pain. The experience of postoperative delirium is associated with the observation of negative postoperative behavioral changes in children within 7 days of hospital discharge. **Conclusions:** The correlation analysis showed a significant positive relationship between CAPD scale and PAED scale. The feature analysis indicated a relationship between CAPD scores and the existence of chronic diseases.

## 1. Introduction

Delirium is commonly seen in hospitalized patients, especially in intensive care units (ICUs). It is associated with deterioration of the patient’s condition. An episode of delirium may lead to serious and negative consequences, such as increased morbidity and mortality among patients. It also causes a prolongation of hospital stay as well as increased days of mechanical ventilation, which results in additional cost of hospitalization. Delirium affects all patients, but the group at significant risk of developing delirium is children. This is due to the intensive nervous system development, which is characteristic of this group [1,2].

Delirium is an acute or subacute condition defined as a disturbance of consciousness, characterized by a sudden onset and a variable course. Postoperative delirium (POD) occurs usually between 1 and 3 days after the operation. However, its occurrence was also reported on postoperative day 0 or 1. In turn, emergence agitation (EA) could appear immediately after recovery from anesthesia, limited mostly to violent agitation and attempts to remove endotracheal tubes, as well as lack of contact. The occurrence of severe arousal upon awakening, e.g., EA should be considered as a strong risk factor of the postoperative delirium, and as such, it was examined by us. What is more, it is sometimes difficult to distinguish between these two stages, keeping in mind their fluctuating nature. It can persist as a short-lived disturbance lasting a few hours or days or develop into a persistent state that endures over time and represents acute mental and physical deterioration. Also referred to as an acute state of confusion, it is a common clinical syndrome in which disturbances of consciousness and a change in cognitive and perceptual functions occur. Delirium prolongs the stay in the hospital by 2–3 days. Its incidence is 23%, but it may increase up to 50% in high-risk surgeries. In the adult general medicine population, incidence is 10–24%. In the case of the surgical population, it affects 37–46%, while occurring in 87% of patients in the ICU. However, in the last case, the range is very vast—it depends on the age of the patients and the type of surgery. In a study conducted by Traube et al., delirium was diagnosed in 25% of patients, and 13% were comatose. Delirium can often be confused with other mental disorders such as schizophrenia, depression, or dementia, which may have similar symptoms. It is also described as a mental disorder that occurs most often during awakening from general anesthesia. The incidence of delirium is high among hospital patients, especially those after surgery or in a critical state. The disorder is not always reversible and quickly passes. One study suggests that only about 4% of patients with delirium symptoms completely resolved before discharge from the hospital. In other cases, it can be even months after the hospital discharge when the symptoms cease. The risk factors of pediatric delirium are as follows: younger age (<5 years old), gender: male, preexisting cognitive impairments or developmental delay, previous delirium, positive family history of delirium, emotional and behavioral problems, certain surgical types like ENT (ear, nose, and throat), and interventions in ocular area [1,2,3,4,5,6,7,8,9].

According to ICD-11 for Mortality and Morbidity Statistics, the susceptibility to delirium in infancy and childhood may be higher than in early or middle adulthood. In childhood, it may also be associated with febrile illnesses and drugs taken (for example, anticholinergics) [10]. Those administered perioperatively opioids, corticosteroids, benzodiazepines, antidepressants, and antiparkinsonian agents (particularly anticholinergic agents) are at high-risk of delirium. The drug’s impact on neurotransmitters influences the development of delirium. The number of drugs administered simultaneously (more than three), psychoactive, and anticholinergic potential are also important. Psychoactive drugs were associated with delirium in 15% to 75% of cases [6]. In addition, delirium developed in up to 80% of ventilated patients [11]. However, some studies report that the prevalence is above 80% of patients, about 90% of whom received benzodiazepines, opioids, or drugs of both groups to reduce the discomfort of intubation. Based on observations and studies, it is suggested that the onset of cognitive impairment is due to the anticholinergic properties of the drugs used [6]. The other non-drug related factors are perioperative blood loss, quality of sleep, diabetes, duration of anesthesia, and surgery. The postoperative factors are bladder catheter or ICU admission. The following environmental factors may exacerbate the incidence of delirium: physical restraints, high noise levels, poor lighting, frequent staff changes [8,12,13,14].

There are two tools that are useful in evaluating pediatric delirium: PAED (Pediatric Anesthesia Emergence Delirium) scale and CAPD scale (Cornell Assessment of Pediatric Delirium). The PAED score was developed in 2004 [15]. It is one of the most widely used scales. It can detect hyperactive delirium in children over 2 years old. It considers the most important features of delirium, such as disturbances of consciousness and changes in cognitive function, as well as related abnormalities concerning psychomotor behavior and emotions. European Society of Paediatric and Neonatal Intensive Care recommends the CAPD scale. It may be used for evaluation for children 0–18 years old, including children with existing developmental delays. It is a modification of the PAED scale. Its overall sensitivity is 94.1%, and it has a specificity of 79.2%. As the patient’s clinical condition worsens, the CAPD score may be useful in diagnosing developing delirium [16,17,18].

Finding the right tools to properly diagnose, monitor, treat, and prevent delirium is important. Delirium occurring in pediatric patients remains an under-recognized and under-studied topic. As a result, there is still a lack of adequate data to indicate its long-term effects. Studies conducted among children are sparse, and the state of knowledge regarding the incidence of delirium is based mainly on extrapolating the literature depicting the phenomenon in adult patients. This study aimed to evaluate the postoperative delirium risk after general anesthesia. This approach was chosen intentionally, as delirium symptoms may appear immediately after anesthesia and can persist into the postoperative period, reflecting a clinical continuum rather than strictly separate entities. Two scales were applied in the evaluation: CAPD and PAED, as these are well-validated, reliable tools specifically designed and extensively utilized for pediatric populations. The CAPD, adapted from the PAED, excels in detecting all delirium subtypes (hyperactive, hypoactive, and mixed) across all ages and developmental stages in critical care settings, with high sensitivity and feasibility for bedside nursing use. The PAED complements it by focusing on emergence delirium post-anesthesia, enabling comprehensive evaluation during the immediate postoperative period when hyperactive symptoms predominate.

To the best of our knowledge, the presented study is the first one in which these two scales were applied and correlated. Besides the delirium, the postoperative behavioral changes were monitored and correlated with the delirium evaluation scores.

## 2. Materials and Methods

### 2.1. Patients Characteristics

A total of 89 patients, who met the criteria of inclusion described in Section 2.2, were enrolled to the study (23 females, 66 males) between 5 months and 14 years old (4.8 ± 3.5), body weight 20.6 ± 11.4 kg. They were patients of the Department of Anesthesiology and Intensive Therapy at Poznan University of Medical Sciences Clinical Hospital. It was a single-center observational cohort study. Data were collected in the years 2022–2024. They underwent surgeries under general anesthesia, which were induced by sevoflurane or propofol. The mean values with standard deviation and median values for ASA (American Society of Anesthesiologists) scale and biochemical parameters are presented in Table 1.

The observational study was approved by the Local Ethical Committee at Poznan University of Medical Sciences (474/20 and 942/23).

### 2.2. Inclusion and Exclusion Criteria to the Study

The patients were included in the study according to the following criteria: age 1 month old to 18 years old, the qualification for general anesthesia, ASA I-III, and the consent of the parent or the guardian for participation in the study. Qualifying patients for the study required the parent or legal guardian to read the necessary information and give written consent for the child to participate in the study.

The exclusion criteria were as follows: lack of consent, ASA > III, allergy to any medicament applied during premedication or/and general anesthesia.

### 2.3. The Study Protocol

The study protocol recorded basic information characterizing the patient, the results of standard laboratory tests performed during the hospital stay, and the degree of sedation according to the Richmond Agitation–Sedation Scale (RASS) scale at the time the patient was admitted to the operating theater. Another element of the protocol was information on premedication, induction, and monitoring of anesthesia and monitoring of vital function assessment parameters and information on the procedure.

General anesthesia, depending on the attending physician’s decision, was either preceded or not preceded by midazolam premedication. For patients who were premedicated, the dose and time of midazolam administration were documented. Propofol or sevoflurane were used for anesthesia induction. Anesthesia was maintained with sevoflurane in a mixture of oxygen and air (MAC during maintenance 0.8–1.2). The analgesic agent used was fentanyl. If muscle relaxation was required, a dose of mivacurium was administered. Before emergence from general anesthesia, patients received analgesic medications such as paracetamol, ibuprofen, or metamizole. After the patient was awakened from general anesthesia until leaving the operating theater, delirium status was assessed in parallel using the CAPD scale and PAED. The CAPD scale had a threshold score for delirium status of ≥9. The PAED scale was used for patients over 2 years old, and the threshold score for delirium status was ≥10. Children under the age of 5 were also assessed for postoperative pain using the Face, Legs, Activity, Cry, and Consolability (FLACC) scale. The threshold score for pain status is ≥5. In order to eliminate variability (bias) in the assessment of the PAED, FLACC, and CAPD scales, internal hospital validation is conducted. The PAED results were missed for 23 patients, and 16 for CAPD—in this case the measurement was not taken into consideration. The missing measurements were not replaced with estimated values—this could artificially reduce variability and potentially distort the results. Instead, we based the analyses solely on observed cases with available data. The final component of the protocol is questionnaires assessing negative postoperative behavioral changes (NPOBCs) after experiencing postoperative delirium, conducted at 1, 7, and 30 days after the child’s discharge from the hospital. The former is the Post-Hospital Behavior Questionnaire (PHBQ), for which no threshold score was established, and the latter is the Parents’ Postoperative Pain Measure (PPPM), for which a score ≥ 6 indicates significant postoperative pain [19,20,21].

### 2.4. The Statistical Analysis

The collected patient data were processed using MS Excel. Statistical analysis was performed using R-Studio software 4.4.0. The Shapiro–Wilk test was used to check the normality of the distribution. For *p*-values > 0.05, a normal distribution is found, while the non-normality of the distribution is assumed when *p* < 0.05. When the requirements for the Student’s *t*-test (equality of groups and/or normality of distribution) were not met, the Mann–Whitney test was used. Correlation analysis was examined using Pearson’s method. The Pearson correlation coefficient was selected due to better utility for strong linear relationships which are common in the literature when different delirium scores are examined as well as when the relation between physiological parameters are referred to patients data like age or body weight [22]. For all statistical tests performed, a confidence level of 0.95 was adopted. For the *t*-student and Mann–Whitney tests, the groups compared are significantly different when the significance level *p* < 0.05. A *p*-value < 0.05 indicates a significant correlation for the Pearson method. In the examined patient group, an evaluation was also conducted to assess the influence on the outcomes of the PAED, CAPD, and FLACC scales of the following factors: premedication before the procedure (yes/no), the use of sevoflurane or propofol for anesthesia induction, and the administration of propofol (yes/no). For the purposes of each analysis, the study group was divided into three sets of two subgroups each. Patients were stratified into groups according to three criteria: (1) premedication with midazolam (Group M: received midazolam; Group NM: no midazolam); (2) agent used for anesthesia induction (Group S: sevoflurane; Group P: propofol); and (3) propofol administration during the procedure (Group RP: received propofol; Group NP: no propofol).

### 2.5. Survey Research

After patients were discharged from the hospital, telephone surveys were conducted with parents of children included in the study to assess negative postoperative behavioral changes. Surveys were implemented at 1, 7, and 30 days after hospital discharge. For this purpose, two questionnaires were used: PHBQ and PPPM. The PHBQ questionnaire was developed to assess behavioral changes in children hospitalized after experiencing postoperative delirium [19]. The PPPM was used to assess postoperative pain in children based on observation by a parent [20].

### 2.6. Feature Analysis and Selection

As part of the feature analysis, we investigated statistical dependencies between selected variables to better understand the dataset’s structure and guide potential feature selection. We examined the association between the CAPD scale and the presence of chronic disease.

For this analysis, the CAPD score was binarized: individuals with values ≥9 were assigned to the high CAPD group (1), and those with scores <9 to the low CAPD group (0). This threshold was chosen based on clinical relevance and the distribution of values in the dataset. The feature analysis was conducted with 59 patients.

A 2 × 2 contingency table was constructed using the binarized CAPD and chronic disease variables (both binary). Due to low expected frequencies in some cells, both the chi-square test and Fisher’s exact test were applied to evaluate the significance of the association.

## 3. Results

### 3.1. The Correlation Diagrams

**Figure 1:**
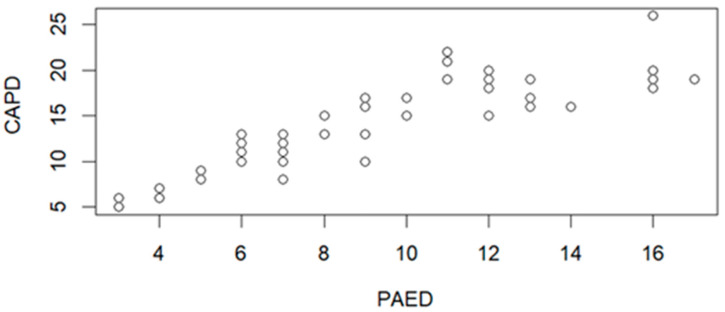
Correlation graph between the PAED delirium rating scale and CAPD (n = 60).

The correlation between CAPD and PAED scale is presented in Figure 1. Statistical parameters R = 0.8745, *p* = 4.636 × 10^−16^, which implies a statistically significant correlation.

**Figure 2:**
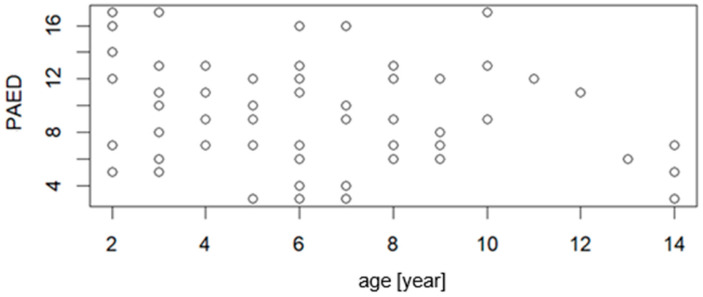
Correlation graph of between PAED scale values and patient age (n = 66).

**Figure 3:**
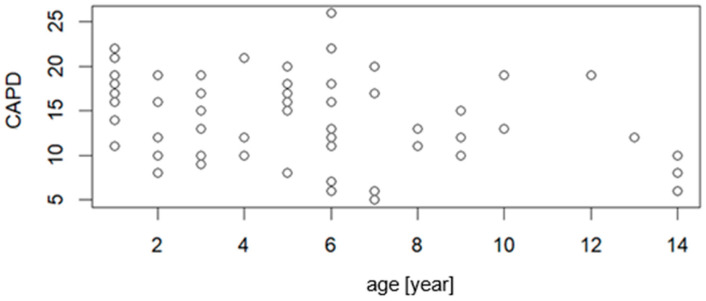
Correlation graph between the relationship of CAPD scale values and patient age (n = 73).

Figure 2 and Figure 3 present the correlation of PAED and CAPD with the age of the patient, respectively. The correlation parameters are R = −0.2636, *p* = 0.0325 for PAED vs. age, and R = −0.3213, *p* = 0.0116 for CAPD vs. age. The low value of correlation coefficients indicated that weak statistically significant correlation was observed.

**Figure 4:**
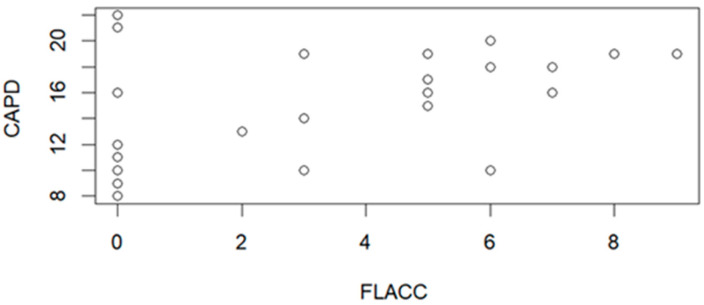
Correlation graph between the relationship of CAPD scale values and FLACC scale values (n = 29).

The CAPD correlation graph with the FLACC scale is presented in Figure 4. The correlation parameters R = 0.4007, *p* = 0.0313, which implies its statistical significance.

**Figure 5:**
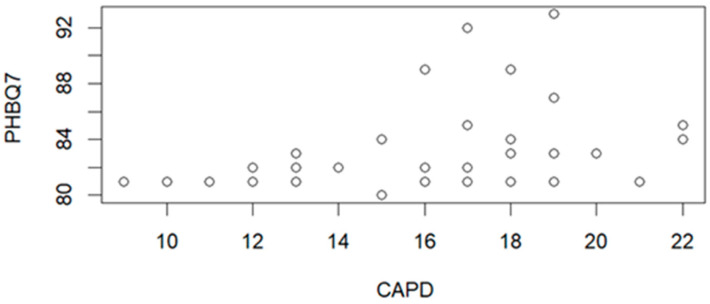
Correlation graph between PHBQ scores on the 7th day (PHBQ7) after leaving the hospital and the values for delirium state on of CAPD scale (n = 53).

The correlation between PHBQ and CAPD scores on the 7th day after leaving the hospital is presented in Figure 5. The correlation parameters (R = 0.3655, *p* = 0.0125) indicate the statistically significant correlation.

**Figure 6:**
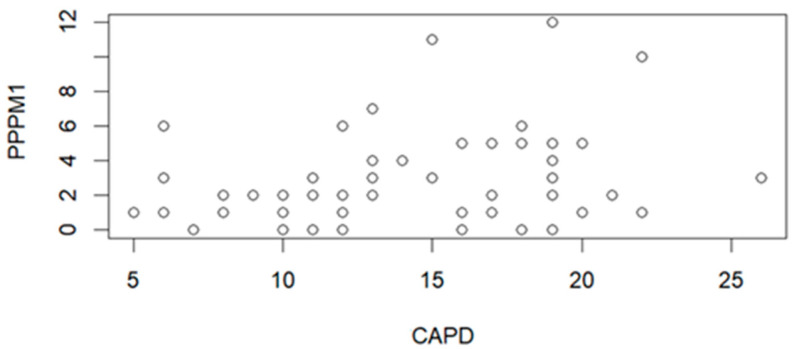
Correlation graph between PPPM1 scores on the 1st day after leaving the hospital and CAPD (n = 57).

The correlation between PPPM and CAPD on the 1st day (PPPM1) after leaving the hospital score is presented in Figure 6. The correlation parameters (R = 0.2749, *p* = 0.0385) indicate the statistically significant correlation.

**Figure 7:**
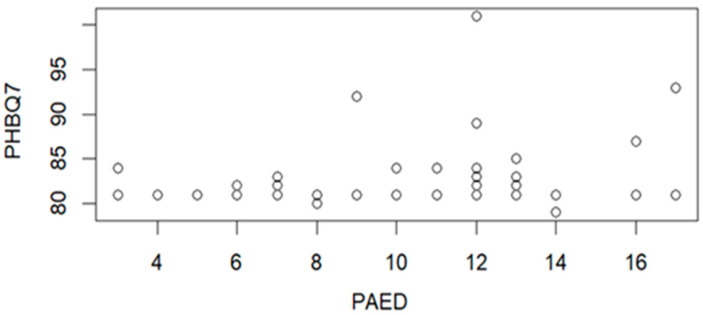
Correlation graph between PHBQ7 and values on the PAED scale (n = 58).

The correlation between PHBQ on 7th day after day after hospital discharge and PAED scale is presented in Figure 7. The correlation parameters (R = 0.2844, and *p* = 0.0305) indicate the statistically significant correlation.

### 3.2. The Statistical Analysis

The results of the statistical analysis between the patients who received premedication with midazolam (Group M) and those who did not receive premedication with midazolam (Group NM) are presented in Table 2. The PAED, CAPD, and FLACC scores were tested. No statistically significant differences were observed.

The results of the statistical analysis between the group of patients in which sevoflurane was used for induction of anesthesia (Group S) and propofol (Group P) are presented in Table 3. The comparison was performed for PAED, CAPD, and FLACC scores. No statistically significant differences were observed.

The results of the statistical analysis between the patients who received propofol during the procedure (Group RP) and the patients who did not receive propofol (Group NP). The results are presented in Table 4. The PAED, CAPD, and FLACC scores were taken into consideration. No statistically significant differences were observed.

### 3.3. Feature Analysis and Selection

The statistical analysis revealed a significant association between the binarized CAPD class and the presence of chronic disease. CAPD scores were dichotomized for this purpose, with values greater than or equal to 9 classified as “high” (1) and values below 9 as “low” (0). This transformation enabled categorical comparison with the binary chronic disease variable.

A chi-square test of independence indicated a statistically significant association between the two variables (χ^2^ = 4.274, *p* = 0.039). Fisher’s exact test was also performed to ensure robustness, particularly given the small sample size in some cells. It confirmed the result, yielding a *p*-value of 0.023. The contingency table is presented in Table 5.

**Figure 8:**
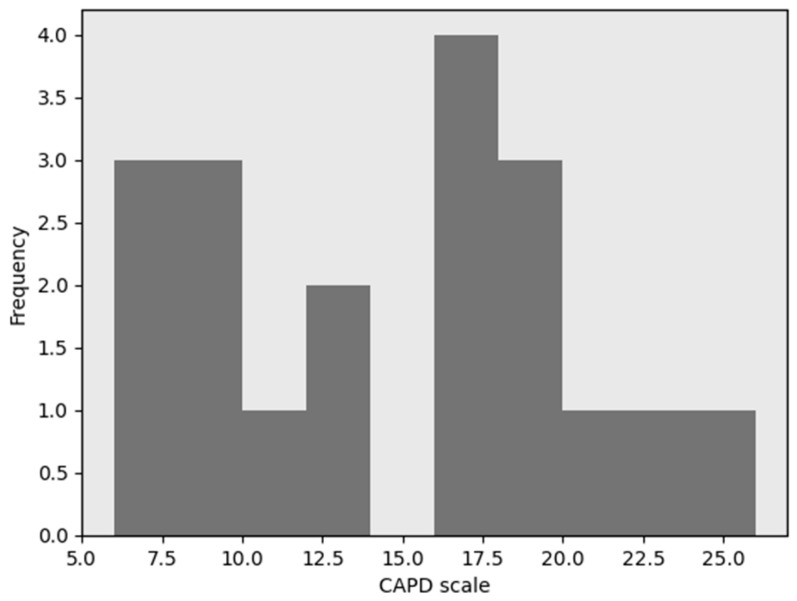
Histogram of the CAPD scale for patients with chronic diseases.

**Figure 9:**
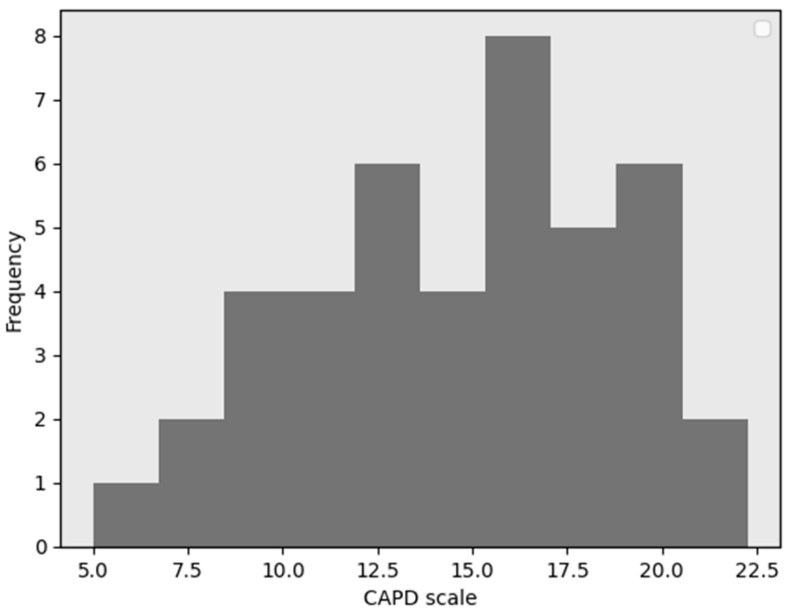
Histogram of the CAPD scale for patients without chronic diseases.

These findings suggest that the presence of chronic illness may be associated withshifts in CAPD classification, with affected individuals showing a comparatively lower proportion in the high-CAPD group. This points to a non-random relationship between physical health status and cognitive-affective regulation. The histograms for CAPD scores for the patients with and without chronic diseases are presented in Figure 8 and Figure 9, respectively.

In cases of patients without chronic diseases, the distribution is bell-shaped (Figure 9). In individuals with chronic diseases, the distribution of scores appears to be altered, suggesting that certain factors specific to this patient population may influence CAPD scale outcomes. In the case of chronic diseases, the histogram distribution is divided into two distinct groups. The separation occurs at a CAPD value of 15 (Figure 8). Therefore, in subsequent analyses, these groups were examined separately. The left group includes values <15, while the right group comprises values ≥15.

## 4. Discussion

The aim of the study was the evaluation of the postoperative delirium risk after general anesthesia in pediatric patients. Two scales were applied: CAPD and PAED. When the delirium was evaluated according to the CAPD scale, the conducted analysis showed that it was observed in 53 patients, which is 60% of the patients’ population in the study. The threshold value for delirium was CAPD ≥ 9 [17]. When the PAED scale was considered, delirium was observed at 35 patients (39% of the population investigated). In this case, the threshold value was PAED ≥ 10 [23]. However, the PAED scale has some limitations—it cannot be applied in evaluating delirium at children below 2 years old [24]. In the study, the highest achieved score was 26 for the CAPD score, while in the case, the CAPD score was 17. In the simultaneous evaluation with these two scores (CAPD and PAED), delirium was noted in 19 patients, which was 21% of the investigated population. Silver et al. [25], in the study concerning the application of the CAPD score for PICU [14] patients, showed that the delirium prevalence was 29%. Moreover, the CAPD score correlated with the DSM-IV criteria, and the accordance was 97%. In the Janssen et al. study [26], delirium was diagnosed in 17% of patients scored with a PAED score. The study also confirmed the utility of the PAED score—it was completed at ca. 94% investigated patients, which is more frequent than DRS-88 (66.9%) or DRS-R-98 (46.8%). The sensitivity of PAED is 91%, and the specificity is 98%. In the abovementioned studies, the delirium was observed when the CAPD score was applied. It was also in accordance with the results obtained in our study. However, in the study of Silver et al., as well as in the study of Janssen et al., the scoring was applied to different patient populations. In our study, the groups were homogeneous, which allows for clearer conclusions. The CAPD score is more sensitive, making it grade of recommendation = A [16].

The correlation analysis between PAED and CAPD revealed a significant correlation between these scales, which indicates the compatibility of these tools in assessing delirium (Figure 1). This implies that both scores can be applied to clinical conditions.

The incidence of delirium may vary. According to Patel et al. the prevalence of pediatric delirium was between 12% and 60% [18]. For patients from the cardiac ICU the delirium was observed in 49% of patients, and the value was similar to that for adults. The delirium developed within the first three days after surgery and lasted 1–2 days. The cardiac bypass surgery increases the risk of delirium incidence [27]. In cases of children between 6 months and 5 years old, the delirium was at 47%. The highest percentage was observed in the group below 2 years old, and it was 56% [28]. Traube et al. also noted that delirium was associated with an age of less than 5 years and an existing developmental delay. The prevalence rate was ca. 21%, and CAPD was applied to the study [17]. In the prospective cohort longitudinal study conducted on ca. 1550 patients of the tertiary care PICU, delirium was diagnosed in 17%. Moreover, in 78% of cases of delirium, it develops within the first three days at the PICU. Delirium occurs frequently also in critically ill children [29]. The interesting conclusions were drawn by Traube et al., who conducted an international study. The prevalence of delirium was 25%, though among children who were treated in the PICU for more than 6 days, it was even 38%. It was evaluated with the CAPD score for delirium observed at the PICU, not the post-operative one [1].

The patient’s age being below 6 years is considered as a risk factor that predisposes the occurrence of delirium. In our study, the correlation of PAED and CAPD with age was weak but statistically significant—*p* < 0.05 (Figure 2 and Figure 3).

Pain is another factor that increases the incidence of delirium. It is a very important issue to distinguish between POD and pain. They may be observed simultaneously. Pain behaviors may resemble and coincide with a condition identified as delirium. Thus, they may be misinterpreted [24]. In the study conducted by Somaini et al., the differentiation between pain and emergence delirium was studied. A total of 512 children were involved in the study with 2048 observations. It occurred that a vast majority of patients (69%) experienced at least one episode of emergence delirium and/or pain. In 15% of patients, it was pain with emergence delirium. It was very important to observe the patient. Such symptoms as ‘no eye contact’ and ‘no awareness of surroundings’ implied the occurrence of emergence delirium. Pain was indicated by ‘abnormal facial expression’, ‘crying’, and ‘inconsolability’ [30]. Our study’s CAPD values were correlated with the FLACC scale (Figure 4). In the case of the FLACC score, the patients older than 5 years old were excluded from the study [21]. The statistical analysis indicated a significant positive correlation between CAPD score and pain level.

The correlation analysis between PHBQ questionnaire scores and CAPD scale values showed a significant correlation for high CAPD values and PHBQ scores on the 1st and 7th day after hospital discharge. In addition, the relationship of PHBQ questionnaire scores on the 1st and 7th day after leaving the hospital to values indicating delirium on the CAPD scale was examined. Patients for whom delirium was observed using the CAPD scale showed higher PHBQ scores 7 days after hospital discharge (*p* = 0.0125) (Figure 5). A significant relationship was also observed when analyzing the relationship between PPPM questionnaire scores on the 1st day after leaving the hospital and CAPD scale values (Figure 6). This means that a high score on the CAPD scale may be associated with higher levels of postoperative pain within 1 day after leaving the hospital (*p* = 0.0385) (Figure 6). No correlation is found between CAPD scale values and PHBQ questionnaire scores on the 30th day after hospital discharge.

Analyzing the correlations between PHBQ questionnaire scores and PAED scale values showed a correlation for scores obtained 7 days after hospital discharge (R = 0.2844, *p* = 0.0305) (Figure 7). No correlation was noted between scores 1 and 30 days after leaving the hospital. The PAED scale scores also showed no correlation between high scores on the postoperative pain measure on the 1st day after leaving the hospital.

The onset of preoperative anxiety is considered as one of the factors contributing to delirium. Before a planned procedure or surgery, it is extremely common to use premedication for preoperative anxiety control [31]. On this basis, the patients included in the study were divided. Group M was of patients who received premedication in the form of midazolam, while Group NM included patients who were not premedicated. The PAED, CAPD, and FLACC scores showed no significant differences between the groups (Table 2). This confirms the accepted theory of the lack of effect of using midazolam as premedication in terms of reducing the risk of delirium.

Another division of patients into two groups was made based on the drug used for the induction of anesthesia. In the case of group S, sevoflurane was used for anesthesia induction, while in group P, propofol was used for induction. It was shown that the analyzed groups did not differ significantly between the results obtained in the PAED, CAPD, and FLACC scales (Table 3). This confirms that there were no significant differences between the use of inhalational and intravenous induction of anesthesia in terms of the risk of development and higher incidence of delirium [32]. However, it needs to be kept in mind that in all cases the anesthesia was maintained with sevoflurane, therefore we cannot categorically exclude the potential differences between sevoflurane and propofol anesthesia in terms of delirium risk.

We assessed the usefulness of propofol in reduction in POD when administered during and/or at the end of surgery. In our study, there were no significant differences between the compared groups in terms of the results obtained in the PAED, CAPD, and FLACC scales (Table 4). Nevertheless, the prophylactic properties of propofol, as an agent for reducing the incidence of delirium, are widely discussed. Some studies indicated that when propofol administered continuously during surgery or as a bolus at the end of anesthesia delivery, it can definitely reduce the risk of delirium [23,31].

The data mining analysis indicated a correlation between the CAPD score and chronic disease. In the case of the adult population (older patients), there is a strong association between chronic diseases and postoperative delirium. Aitken et al. indicated that cognitive impairment increases delirium risk by 9.8-fold in vascular surgery patients [33]. The other factors, depression and hypertension, are also identified as key prognostic factors [33,34]. In cases of cardiosurgical patients, the cardiovascular comorbidities (atrial fibrillation, cerebrovascular disease) and metabolic disorders (diabetes mellitus) significantly elevate the risk of delirium in cardiovascular patients [34]. In the case of children, the correlation was less established. However, different mechanisms were suggested. Lin et al. [35] indicated that developmental delays emerge as the primary chronic risk factor. Chronic neurological conditions may interact with such factors as pain or specific surgeries (e.g., otorhinolaryngology procedures) [35]. On the other hand, this finding should be interpreted with caution. Variables such as age distribution, baseline neurodevelopmental status, type and severity of chronic conditions, and exposure to sedative or psychoactive medications were not controlled for in the present analysis and may have substantially influenced CAPD scores. In particular, older age and medication burden are well-established contributors to altered mental status and could distort the observed relationship. Additionally, the heterogeneous nature of chronic diseases within the cohort may mask divergent effects of specific conditions on cognitive-affective regulation. Therefore, this association should be considered exploratory, and future studies with larger samples and multivariable adjustment are required to clarify the independent contribution of chronic disease to CAPD outcomes.

The feature analysis identified a specific subgroup of patients within the CAPD < 9 group. These individuals potentially use medications that mitigate immune system reactions (e.g., antihistamines or immunosuppressants), primarily including allergy sufferers and one post-transplant patient. Within the allergy subgroup, patients potentially taking such medications are those diagnosed with atopic dermatitis and allergies to pollen, grasses, birch, molds, dust, and mites. We observed that patients potentially receiving immunomodulatory medications (such as antihistamines or immunosuppressants) were predominantly found in the group with lower CAPD values. This may suggest that the use of such drugs is associated with a reduced level of the measured marker. Due to the limited sample size and the lack of data regarding actual medication intake, this finding should be considered preliminary, and the results from the analysis that links immunomodulatory medications with protective effect against delirium should be treated with caution. The observed clustering of patients with conditions typically requiring immunomodulatory treatment in the lower CAPD range should therefore be regarded as hypothesis-generating. Nevertheless, it highlights a potentially important direction for future research incorporating pharmacological factors. The meta-analysis conducted by Kim et al. [36] indicated that the application of non-steroidal anti-inflammatory drugs reduced the incidence of postoperative delirium as well as the use of the opioids and severity of pain. The association of postoperative delirium with cytokines and inflammatory biomarkers was confirmed by Mosharaf et al. [37]. This study supported the hypothesis that inflammation plays a significant role in the pathogenesis of delirium. The results from affinity analysis in our study confirmed it—the patients taking immunomodulatory drugs were found in the group with the lower CAPD score. Future studies incorporating comprehensive pharmacological data are needed to determine whether modulation of inflammatory responses influences delirium-related outcomes.

The study’s use of validated delirium assessment tools (CAPD and PAED) and a homogeneous pediatric population supports the generalizability of the findings within similar clinical settings. The observed delirium prevalence aligns with previously reported ranges, confirming external consistency. Correlations with pain and postoperative behavioral measures further validate clinical relevance. However, limitations such as the PAED scale’s restriction in children under 2 years and differences in ICU populations suggest caution when extending results to younger infants or specialized units. Overall, the findings are applicable to pediatric patients undergoing general anesthesia but should be contextualized based on patient age and care environment. Additionally, the data mining analysis linking CAPD scores with chronic disease provides insight into the complex risk factors for delirium. While in adults, strong associations exist between delirium and chronic conditions such as cognitive impairment, depression, hypertension, and cardiovascular/metabolic comorbidities, these correlations in children are less established. Developmental delays and chronic neurological conditions appear as primary chronic risk factors in pediatric populations, interacting with pain and specific surgeries. The identification of a subgroup of patients potentially using immunomodulatory medications—with lower CAPD scores—suggests a possible protective pharmacological effect, aligned with meta-analyses showing anti-inflammatory drugs reduce delirium incidence. Although preliminary due to limited sample size and medication data, this points to important directions for future research integrating pharmacological factors in delirium risk assessments.

### Limitations of the Study

This study has some limitations. Firstly, this is a single-centered study, which resulted in the moderate number of patients enrolled in the study. Secondly, some of the PAED and CAPD values were not available—these cases were not taken into consideration in the analysis. Thirdly, assessment timing was limited to certain postoperative days, potentially missing nocturnal or delayed delirium episodes and resulting in underestimation of prevalence.

## 5. Conclusions

The compatibility and concurrent association of these instruments in evaluating pediatric delirium were confirmed by the correlation analysis that showed a significant positive relationship between CAPD scale and PAED scale. This result implied the clinical usefulness of both measures for accurate assessment of delirium in children, especially in critically ill children as well as after surgery. An investigation using feature analysis discovered a relationship between CAPD scores and the existence of chronic diseases, which agrees with findings in adults associating cognitive decline, depression, hypertension, and certain cardiovascular diseases with a higher risk of delirium. In children, the presence of certain developmental delays along with chronic neurologic illnesses seem to be the main risk factors. Moreover, patients likely to be on some immunomodulatory medications like antihistamines or immunosuppressants are associated with lower CAPD scores, indicating a possible protective role these medications may have on delirium, though this observation is weakened due to small sample size. Feature analysis is a useful tool for data analysis aimed at identifying prognostic factors (covariates) beyond those originally assumed in the study that could be applied in machine learning analysis [38].

## Figures and Tables

**Table 1:** The results for ASA and biochemical parameters in the investigated group (n = 89).

Parameter	Mean ± SD	Median
ASA	1.71 ± 0.63	2.00
Hb [g/dL]	12.65 ± 0.93	12.70
Htk [%]	36.04 ± 2.84	35.90
Ery [T/L]	4.69 ± 0.40	4.69
Leu [G/L]	8.28 ± 2.57	8.11
PLT [G/L]	328.88 ± 72.33	321.00
K [mEq/L]	4.51 ± 0.41	4.48
Na [mEq/L]	138.96 ± 3.20	139.00

**Table 2:** Division of patients depending on the use of premedication according to PAED, CAPD, and FLACC (group M—patients who received midazolam as premedication, group NM—patients who did not receive midazolam as premedication).

Group M				Group NM				
Minimum	Maximum	Median	Mean	Minimum	Maximum	Median	Mean	*p*-Value
PAED score								
3	17	10	9.9	7	12	9.5	9.5	0.8911
CAPD score								
5	26	16	14.6	10	19	13.5	14	0.7939
FLACC score								
0	9	5	3.9	0	9	3.0	3	0.5328

**Table 3:** Division of patients depending on the drug used for induction of anesthesia according to PAED, CAPD, and FLACC (group S—induction of anesthesia with sevoflurane, group P—induction of anesthesia with propofol).

Group S				Group P				
Minimum	Maximum	Median	Mean	Minimum	Maximum	Median	Mean	*p*-Value
PAED score								
3	17	10	10	5	13	9	9.2	0.5111
CAPD score								
5	26	15	14.4	8	22	16.5	15.4	0.4648
FLACC score								
0	9	5	4.1	0	6	2	2.2	0.1636

**Table 4:** Division of patients based on propofol application during the procedure according to PAED, CAPD, and FLACC (group RP—patients who received propofol during procedure, group NP—patients who did not receive propofol during procedure).

Group RP				Group NP				
Minimum	Maximum	Median	Mean	Minimum	Maximum	Median	Mean	*p*-Value
PAED score								
3	17	10.5	9.8	3	17	10	9.9	0.8954
CAPD score								
6	22	16	14.3	5	26	15.5	14.9	0.6145
FLACC score								
0	9	5	4.2	0	9	3	3.4	0.4031

**Table 5:** A 2 × 2 contingency table for χ^2^-test for chronic disease.

Chronic Disease	CAPD Class: False	CAPD Class: True
No (0)	2	39
Yes (1)	5	13

## Data Availability

The raw data supporting the conclusions of this article will be made available by the authors on reasonable request.

## Abbreviations

ASA	American Society of Anesthesiologists
CAPD	Cornell Assessment of Pediatric Delirium
EA	Emergence agitation
FLACC	Face, Legs, Activity, Cry and Consolability
ICU	Intensive Care Unit
NPOBC	Negative Postoperative Behavioral Changes
PAED	Pediatric Anesthesia Emergence Delirium
PHBQ	Post-Hospital Behavior Questionnaire
PICU	Pediatric Intensive Care Unit
POD	Postoperative delirium
PPPM	Parents Postoperative Pain Measure
RASS	Richmond Agitation-Sedation Scale
